# Supplementary material for: Has the educational use of digital technologies changed after the pandemic? A longitudinal study
Source: PLoS One. 2024 Dec 12;19(12):e0311695. doi: 10.1371/journal.pone.0311695 (PMC11637279; doi:10.1371/journal.pone.0311695)
Supplement: Attachment — Submitted filename: Response to Reviewers.docx [file pone.0311695.s002.docx]

Dear Editor,

We would like to express our sincere gratitude to you and the reviewers for your valuable feedback on our manuscript titled “Has the Educational Use of Digital Technologies Changed After the Pandemic? A Longitudinal Study.” Below, we have outlined the suggestions provided by both you and the reviewers:

**Editor’s Suggestions:**

1. **Method:**
   - It was suggested to delve deeper into the statistical tests performed, the appropriateness of the data, and its evaluation.

*Thank you very much for your suggestion. In section 2.3, "Analysis of the results," we have tried to delve deeper into the statistical tests performed as indicated.*

2. **Discussion:**
   - The editor advised discussing the limitations of the K-means analysis and what these limitations imply for the findings.

*Thank you very much for the recommendation. In section 4.1, "Limitations of the study," we have addressed the limitations of the K-means analysis.*

- It was also recommended to ensure that the findings and conclusions are aligned with the results, without offering any conclusions not supported by the data.

*We greatly appreciate your suggestion. We have included a few adjustments on page 22 of the manuscript to improve readability.*

3. **Other Recommendations:**
   - We were asked to clarify the role of the funders in the study, and if they had no role, to explicitly state that in the cover letter.

*As the editor recommended, we have added the following statement: "The funders had no role in study design, data collection and analysis, decision to publish, or preparation of the manuscript."*

- We were informed that funding information should appear in the Cover Letter and not in other sections of the manuscript.

*We have removed the funding information from the manuscript and included it in the cover letter.*

- The editor suggested providing a non-author contact for data access requests to ensure long-term availability of the data.

*We have added an institutional link where both the questionnaire used and the dataset can be found, which will make it easier for readers to replicate the data:* [*https://doi.org/10.21950/VYBSEP*](https://doi.org/10.21950/VYBSEP)

- The inclusion of separate legends for each figure in the manuscript was requested.

*We have included separate legends for each figure as recommended.*

- We were also advised to review the references, and any changes made should be noted in the letter to the editors and reviewers.

*We have added the following references to the manuscript, but no changes have been made to the previous references:*

- *Valverde-Berrocoso J, Fernández-Sánchez MR, Revuelta Dominguez FI, Sosa-Díaz MJ. The educational integration of digital technologies pre-Covid-19: Lessons for teacher education. PLoS One. 2021;16(8).* [*https://doi.org/10.1371/journal.pone.0256283*](https://doi.org/10.1371/journal.pone.0256283)
- *Peng R, Abdul Razak R, Halili SH. Factors influencing in-service teachers’ technology integration model: Innovative strategies for educational technology. PLoS One. 2023;18(8).* [*https://doi.org/10.1371/journal.pone.0286112*](https://doi.org/10.1371/journal.pone.0286112)
- *Vasuthevan K, Vaithilingam S, Ng JWJ. Academics’ continuance intention to use learning technologies during COVID-19 and beyond. PLoS One. 2024;19(1).* [*https://doi.org/10.1371/journal.pone.0295746*](https://doi.org/10.1371/journal.pone.0295746)
- *Ahmed, M, Seraj, R, Islam, SMS. The k-means algorithm: A comprehensive survey and performance evaluation. Electronics.2020;  9(8): 1-12.* [*https://doi.org/10.3390/electronics9081295*](https://doi.org/10.3390/electronics9081295)

**Reviewer 1’s Suggestions:**

Reviewer 1 expressed full satisfaction with the manuscript.

*We sincerely thank Reviewer 1 for their positive feedback and full satisfaction with our work. We are pleased that the manuscript meets the high standards expected, and we appreciate their recognition of the effort and rigor that went into this research.*

**Reviewer 2’s Suggestions:**

1. **Conclusions:**
   - The reviewer suggested that while the conclusions are consistent with the results, the discussion could be enriched by including references related to similar studies, particularly those published in PLOS ONE concerning teaching practices with ICT before the lockdown.

*As indicated to the editor, we have added a series of references published in PLOS ONE that help justify the discussion of the results obtained.*

2. **Ethics:**
   - The reviewer noted that although the Data Availability Statement indicated that all data were fully accessible, there was no clear method to access these data, including the complete questionnaire. The reviewer emphasized the importance of making the full instrument available for better understanding of the results.

*We have added an institutional link where both the questionnaire used and the dataset can be found, which will make it easier for readers to replicate the data:* [*https://doi.org/10.21950/VYBSEP*](https://doi.org/10.21950/VYBSEP)

3. **Method:**
   - The reviewer pointed out that while information about the reliability of the questionnaire was provided, information regarding the validity of the instrument was missing and should be included.

*We greatly appreciate the reviewer's suggestion. In section 2.1, "Task and procedure," we have added information regarding the validity of the instrument.*

We appreciate the detailed feedback provided, which will help us to refine and improve our manuscript.

Yours faithfully,

The authors of the manuscript.
